# “Head to Head”: pneumocephalus as a complication of soccer

**DOI:** 10.1186/1865-1380-6-46

**Published:** 2013-12-19

**Authors:** Eamon C Francis, Eimhear Quinn, John Ryan

**Affiliations:** 1 Department of Emergency Medicine, St Vincent’s University Hospital, Elm Park, Dublin 4, Ireland

## Abstract

**Background:**

Pneumocephalus is uncommon in craniofacial trauma and a rare occurrence in non-contact sports. It may be asymptomatic or present with signs of increased intracranial pressure and the majority of cases will resolve with conservative management. However, there should be a high index of clinical suspicion to recognise, diagnose, and manage it appropriately, as complications may be fatal.

**Findings:**

To our knowledge, this is the first case report of pneumocephalus secondary to a “minor” head injury during a soccer match.

**Conclusion:**

We outline the management of this condition and highlight signs that should generate a high index of suspicion.

## Findings

### Introduction

Pneumocephalus, also known as intra-cerebral aerocele or pneumatocele, is defined as the presence of gas within any of the intracranial compartments of the cranial vault [1]. It is usually associated with disruption of the skull after head and facial trauma, tumours of the skull base, or following neurosurgery or otorhinolaryngological procedures, and can rarely occur spontaneously [2]. Clinical presentations typically include headaches, nausea, vomiting, seizures, dizziness, and a depressed neurological status [3].

We herein report on a case of a 17-year-old male who presented with asymptomatic pneumocephalus associated with craniofacial trauma after a soccer match. The pathophysiological mechanisms, diagnosis, and management are discussed with reference to the current literature.

### Case

We report the case of a 17-year-old male who self-presented to the minor injuries section of our emergency department after a soccer match. He reported a right sided facial swelling and pain following a mid-air collision with another player two hours earlier. He also reported having had an episode of epistaxis, and after blowing his nose, he was unable to actively open his right eye.

**Figure 1:**
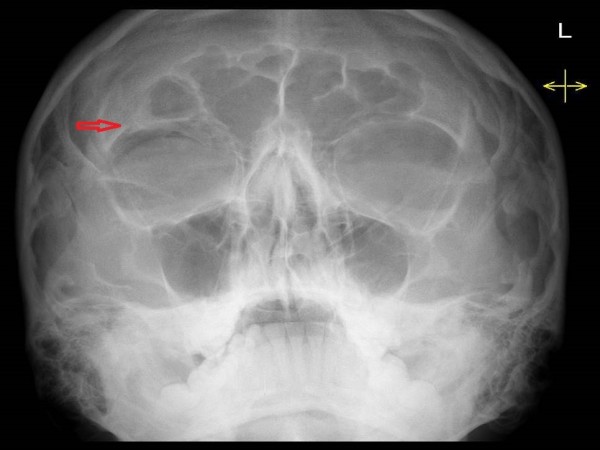
XR facial bones.

**Figure 2:**
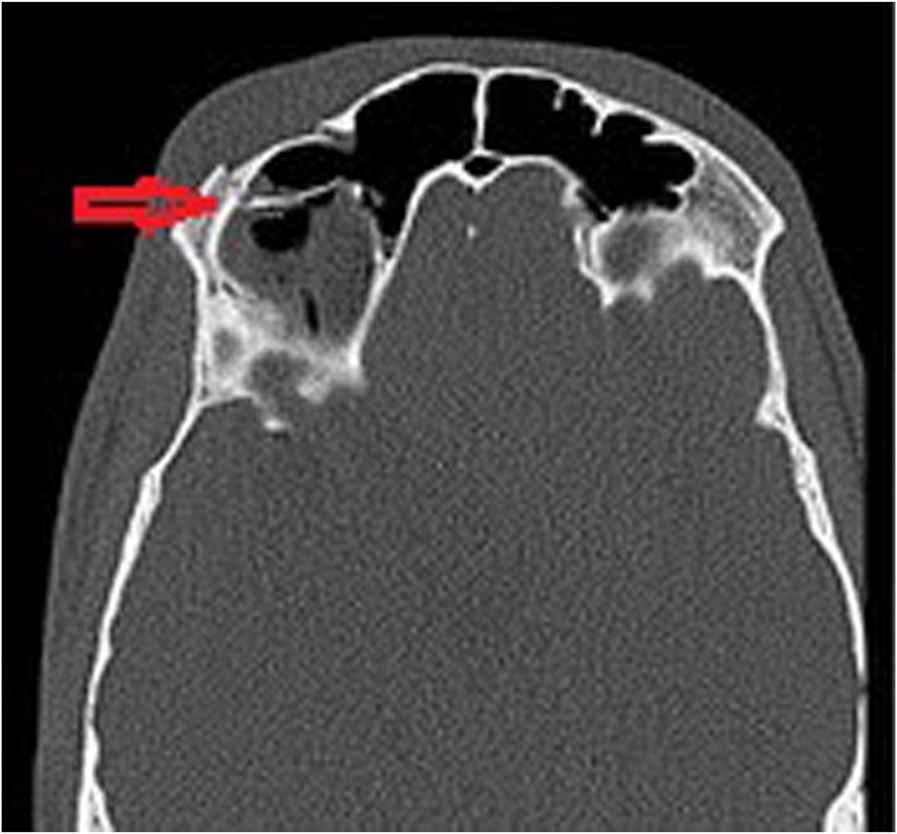
CT facial bones.

**Figure 3:**
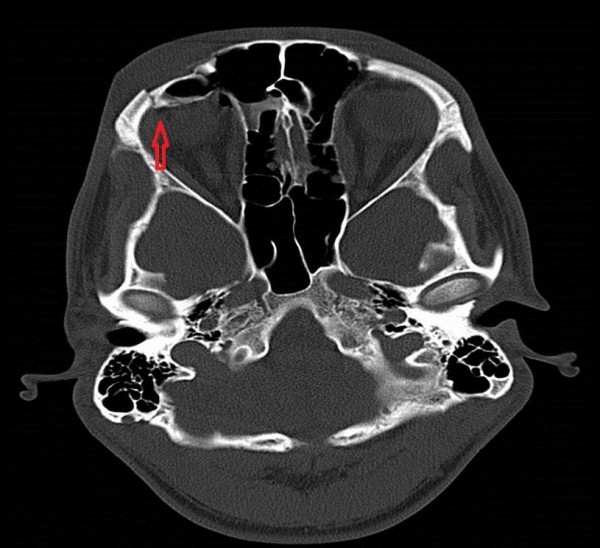
CT brain.

On examination, he had no neurological deficit and his Glasgow Comma Score was 15/15. He had a superficial laceration above his right eyebrow and a palpable depression in the right supraorbital region. He was actively unable to open his right eye and his vision was blurred. He had no diplopia or nystagmus. There were no clinically evident signs of basal skull fracture. Plain radiographs were obtained and demonstrated a displaced fracture of the supraorbital rim (Figure 1). CT of the facial bones confirmed a fracture of the anterior wall of the frontal sinus and showed pneumocephaly (Figure 2). A CT of the brain was performed confirming the presence of free air and ruled out any intra- or extra-axial haemorrhage (Figure 3).

Antibiotic prophylaxis was administered intravenously and opinions from the neurosurgery, maxillofacial surgery, and ophthalmology departments were sought. He was neurologically observed for 12 hours in our clinical decision unit after which a consensus was reached that no acute intervention was required. He was transferred to the maxillofacial surgery department the following morning and was administered a broad-spectrum oral antibiotic for one week, after which he was assessed for surgical repair of the injury.

### Discussion

The first description of intracranial pneumocephalus provided by Thomas in 1866 was discovered during the autopsy of a trauma patient [4] and the term was later coined by Wolf in 1914 [5].

Pneumocephalus is a rare complication of non-contact sports, such as soccer, with the exception of diving, where it is more commonly seen. Although typically asymptomatic in patients, pneumocephalus of sufficient volume can manifest in signs of increased intracranial pressure analogous to those of a cerebral bleed, such as headaches, nausea, vomiting, confusion, hemiparesis, and abducens nerve palsy [3,6].

There are two mechanisms implicated in the generation of pneumocephalus, “ball valve” and “inverted bottle”. The “ball valve” theory suggests that positive pressure via nose blowing, coughing, and Valsalva manoeuvers, as demonstrated in our case, forces air through the cranial defect, which may resist the outflow of air. Significant resistance may lead to a tension pneumatocele [7]. In the “inverted bottle” mechanism, drainage of cerebrospinal fluid creates a negative intracranial pressure gradient, which is negated by the entry of air [8]. The amount of air is independent of the size of the defect, but smaller defects are more easily sealed by blood clots or granulation, allowing for gradual reabsorption and spontaneous resolution.

Plain films can be used to demonstrate large collections of air and facial bone fractures. However, this has largely been superseded with the advent of CT. Air has a Hounsfield coefficient of −1000 on CT, which enables the sensitive and specific detection of even minute quantities of intracranial air; 0.5 mL of air can be visualized with CT in contrast to 2 mL, which is the minimum that can be seen with plain films [9]. However, CT is only performed in patients with clinical indications and in combination with local guidelines.

### Conclusions

Emergency medicine physicians should beware of triaged “minor” cranio- or maxillofacial trauma. There should be a high index of clinical suspicion of complications, such as pneumocephalus, that may arise from seemingly “minor” trauma. Pneumocephalus may be asymptomatic or present as a space-occupying lesion. It requires prompt recognition and management to prevent unwanted morbidity and mortality such as meningitis or a cerebrospinal fluid fistula.

Physicians should be aware of this clinical entity and consider it in their differential diagnosis. CT is the most useful diagnostic tool and should be considered in accordance with the patient’s clinical examination and local guidelines in patients with facial trauma. These injuries require a multidisciplinary team approach and are best managed in facilities with specialist cover onsite.

### Consent

Written informed consent was obtained from the patient’s guardian/parent/next of kin for the publication of this report and any accompanying images.

## Competing interests

The authors declare that they have no competing interests.

## Authors’ contributions

EC wrote article, EQ reviewed literature, JR supervised and reviewed manuscript. All authors read and approved the final manuscript.

## References

[B1] Marsh AG Barker A Campbell A Stoica S Nkere U An unusual case of cerebrospinal fluid leak and pneumocephalus following a lumbar stab injury Injury Extra 2008 6 7 250 252 10.1016/j.injury.2008.01.019

[B2] Schirmer CM Heilman CB Bharwaj A Pneumocephalus: case illustrations and review Neurocrit Care 2010 6 1 152 158 10.1007/s12028-010-9363-0 20405340

[B3] Markham JW The clinical features of pneumocephalus based upon a survey of 284 cases with report of 11 additional cases Acta Neurochir (Wien) 1967 6 1 78 10.1007/BF01401900 6032371

[B4] Thomas L Du pneumatocele du crane Arch Gen Med (Paris) 1866 6 34 55

[B5] Wolff E Luftansammlung im rechten Seitenventrikel des Gehirns (Pneumozephalus) Munch Med Wochenschr 1914 6 899

[B6] Standefer M Bay JW Trusso R The sitting position in neurosurgery: a retrospective analysis of 488 cases Neurosurgery 1984 6 649 658 10.1227/00006123-198406000-00001 6462398

[B7] Delgaudio JM Ingley AP Treatment of pneumocephalus after endoscopic sinus and microscopic skull base surgery Am J Otolaryngol 2010 6 4 226 230 10.1016/j.amjoto.2009.02.012 20015750

[B8] Lunsford LD Maroon JC Sheptak PE Albin MS Subdural tension pneumocephalus. Report of two cases J Neurosurg 1979 6 525 527 10.3171/jns.1979.50.4.0525 423011

[B9] McIntosh BC Strugar J Narayan D Traumatic frontal bone fracture resulting in intracerebral pneumocephalus J Craniofac Surg 2005 6 461 463 10.1097/01.SCS.0000157249.31826.B7 15915116

